# PF-D-Trimer, a protective SARS-CoV-2 subunit vaccine: immunogenicity and application

**DOI:** 10.1038/s41541-023-00636-8

**Published:** 2023-03-15

**Authors:** Zhihao Zhang, Jinhu Zhou, Peng Ni, Bing Hu, Normand Jolicoeur, Shuang Deng, Qian Xiao, Qian He, Gai Li, Yan Xia, Mei Liu, Cong Wang, Zhizheng Fang, Nan Xia, Zhe-Rui Zhang, Bo Zhang, Kun Cai, Yan Xu, Binlei Liu

**Affiliations:** 1 National “111” Center for Cellular Regulation and Molecular Pharmaceutics, Key Laboratory of Fermentation Engineering (Ministry of Education), Hubei Provincial Cooperative Innovation Center of Industrial Fermentation, Hubei Key Laboratory of Industrial Microbiology, Hubei University of Technology, Wuhan, Hubei China; 2 Wuhan Binhui Biopharmaceutical Co., Ltd, Wuhan, China; 3 Institute of Health Inspection and Testing, Hubei Provincial Centre for Disease Control and Prevention (Hubei CDC), Wuhan, Hubei China; 4 Nova Biologiques Inc. Montréal, Québec, Canada; 5 School of Pharmacy, Hubei University of Science and Technology, Xian Ning, China; 6 Wuhan Institute of Virology, Chinese Academy of Sciences, Wuhan, China

**Keywords:** Recombinant vaccine, Vaccines

## Abstract

The COVID-19 pandemic, caused by the SARS-CoV-2 virus, has had and continues to have a significant impact on global public health. One of the characteristics of SARS-CoV-2 is a surface homotrimeric spike protein, which is primarily responsible for the host immune response upon infection. Here we present the preclinical studies of a broadly protective SARS-CoV-2 subunit vaccine developed from our trimer domain platform using the Delta spike protein, from antigen design through purification, vaccine evaluation and manufacturability. The pre-fusion trimerized Delta spike protein, PF-D-Trimer, was highly expressed in Chinese hamster ovary (CHO) cells, purified by a rapid one-step anti-Trimer Domain monoclonal antibody immunoaffinity process and prepared as a vaccine formulation with an adjuvant. Immunogenicity studies have shown that this vaccine candidate induces robust immune responses in mouse, rat and Syrian hamster models. It also protects K18-hACE2 transgenic mice in a homologous viral challenge. Neutralizing antibodies induced by this vaccine show cross-reactivity against the ancestral WA1, Delta and several Omicrons, including BA.5.2. The formulated PF-D Trimer is stable for up to six months without refrigeration. The Trimer Domain platform was proven to be a key technology in the rapid production of PF-D-Trimer vaccine and may be crucial to accelerate the development and accessibility of updated versions of SARS-CoV-2 vaccines.

## Introduction

The appearance of the SARS-CoV-2 virus in 2019 quickly became a major public health problem with the rapid spread of the COVID-19 pandemic knowing no borders^1^. Like most enveloped RNA viruses, SARS-CoV-2 uses a trimeric surface protein, the spike (S) protein, when infecting a host cell. S protein is responsible for the attachment by binding to a cellular receptor, hACE2^2^ and then mediates viral entry by membrane fusion^3^. Several research results and available vaccines have already demonstrated the importance of the spike protein as an ideal target for vaccine development^4^. Current SARS-CoV-2 vaccines can be categorized into four different classes, summarily: nucleic acids, RNA or DNA, which encode part of the genetic information of the virus; inactivated vaccines which consist of a virus being physically treated to render it incapable of producing disease; viral vector vaccines, for example, adenovirus with limited replication capacity, which encodes part of the SARS-CoV-2 genome to introduce it into a host cell; recombinant protein subunit vaccines, which do not use viral genetic material, but rather full-length viral proteins or fragments thereof, either packaged or not in nanoparticles for better delivery and uptake by cells responsible for immunity^5,6^. It has been known for several years that major epitopes of the S protein of coronaviruses only exist in its trimeric form, and are so trimer restricted^7^. Recently published research results also support the concept that the trimeric form of the S protein adopts a conformation containing important vaccine epitopes^8^. As demonstrated in a recent publication, low titers of neutralizing antibodies are associated with SARS-CoV-2 Delta breakthrough infections in vaccinated patients^9^. Even if the Delta variant maintains its cryptic circulation, there is evidence for SARS-CoV-2 Delta and Omicron co-infections and recombination^10–12^. Sera from unvaccinated or vaccinated Delta-wave intensive care unit (ICU) patients strongly neutralize Omicron BA.4/5 and BA.2.12.1^13^.

PF-D-Trimer and PF-W-Trimer (PF-Trimers) are subunits SARS-CoV-2 vaccine candidates, consisting of the recombinant S-glycoprotein from the Delta and original WA1 variants in their prefusion form^14^ and trimerized by fusion with our proprietary Trimer Domain (TD). TD is a fragment from the hemagglutinin long alpha helix covalently linked by disulfide bonds located according to their natural intermolecular proximity in the heptad repeat. In addition to the Spike protein, Trimer Domain has already been successfully used for the stabilization, trimerization, expression, and purification of soluble SARS-CoV-2 RBD and S1 and influenza hemagglutinin H7^15^. We demonstrate that PF-D-Trimer adjuvanted with alum and CpG 1018 induces a strong immune response in the form of circulating and neutralizing antibodies against the original WA1 virus, as well Delta and different Omicron variants, including BA.2.2 and BA.5.2. The formulation also induces a cellular immune response in animal models and protect K18-hACE2 H11 transgenic mice in a homologous challenge study. These results support our vaccine strategy of using the Delta variant S protein as an antigen. In addition, we describe here the use of our TD platform, which not only allows the stabilization of the S protein in a trimeric form, but also its simplified and rapid one-step purification by immunoaffinity, allowing the development of a streamlined chemistry manufacturing and control (CMC) strategy. Formulated PF-D-Trimer remains stable when kept at 25 °C for up to six months. It is, therefore, foreseeable that local distribution could be achieved without refrigeration, thus facilitating SARS-CoV-2 vaccination in countries where it would be difficult to maintain an adequate cold chain.

## Results

### High level expression, purification, and characterization of PF-D-Trimer and PF-W-Trimer from bioreactor cultures

**Fig. 1:**
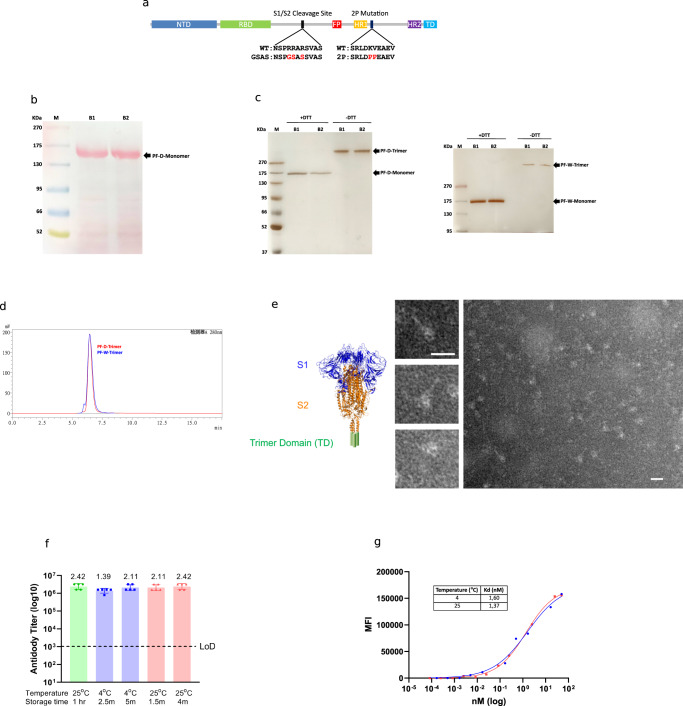
Expression, purification, and characterization of the PF-trimers. **a** Schematic representation of the S ectodomain of SARS-CoV-2 Trimer Domain fusion proteins. **b** Analysis by reducing SDS-PAGE with Ponceau S staining of high-level expression of PF-D-Trimer from CHO cells in bioreactor cultures over a period of 11 days. Samples from two batches (B1, B2) were analyzed side by side. Each well contains 16 μL of CHO cells culture supernatant. **c** The PF-Trimers are disulfide bond-linked homotrimeric proteins as analyzed by SDS-PAGE with silver staining under reducing (+DTT) and non-reducing (-DTT) conditions. Samples from two purification batches (B1, B2) were analyzed side by side. Each well contains 1 µg of PF-Trimers as quantified by molar absortivity at 280 nm. Each well of a gel derives from the same experiment and has been treated in parallel. **d** SEC-HPLC analysis of the immunoaffinity purified PF-D-Trimer (red) and PF-W-Trimer (blue) showing a MW of approximately 525 kDa and a purity of more than 99%. **e** PF-D-Trimer schematic diagram and representative negative-stain EM images with homotrimeric S protein in the prefusion conformation. The images on the left are partial enlargements of the image on the right as indicated with the black arrows. The sample contains assembled spike trimers. Scale bar 20 nm. **f** PF-D-Trimer stability assays. Antibody titers for sera obtained from Sprague-Dawley rats 28 days following immunization with 10 µg PF-D-Trimer formulated with alum plus CpG 1018 that had been stored at 4 °C or 25 °C for up to five months, compared to freshly formulated PF-D-Trimer (left, green circles). Points represent individual animal; bars indicate geometric mean titers (GMT) responses (with geometric standard deviation). Dotted lines indicate the limit of detection, LOD; 1 × 10^5^. **g** Dissociated PF-D-Trimer cellular binding using HEK293/hACE2 cells. The binding assays were performed as described in “Methods”. MFI, Median Fluorescence Intensity. ● Formulation stored at 4 °C for six months, ■ Formulation stored at 25 °C for six months.

PF-D-Trimer and PF-W-Trimer mimic the native trimeric prefusion structure of the S protein, as found on the virus surface, and consists of the complete ectodomain of the S protein of the SARS-CoV-2 Delta (PF-D-Trimer) and original WA1(PF-W-Trimer) variants fused C-terminally to the TD trimerization domain (Fig. 1a). A high-level production process was developed following the transfection of CHO cells with an expression vector containing a CHO codon-optimized cDNA encoding the PF-Trimers, the selection of the best performing clones and process optimizations. This process makes it possible to achieve production levels of more than 500 mg/L of Trimers as a secreted protein in the culture medium. The expression process is robust as demonstrated in the side-by-side analysis of two different batches of PF-D-Trimer by SDS-PAGE, which yield nearly identical expression levels (Fig. 1b). Reducing and non-reducing SDS-PAGE analysis demonstrated that the purified PF-Trimers are self-associating homotrimers stabilized by interchain disulfide bonds (Fig. 1c). Side-by-side analysis of two purification batches of PF-D-Trimer demonstrates the reproducibility of the purification process, the two batches having similar apparent levels of purity after silver staining (Fig. 1c). Under reducing conditions, the PF-Trimers appears as a single form with a molecular weight of around 170 kDa. Under non-reducing conditions, the PF-Trimers appears as a single high molecular weight form, thus demonstrating that the protein is not cleaved by proteases produced by CHO cells (Fig. 1c). The PF-Trimers eluted from the one-step α−TD trimerization domain monoclonal antibody immunoaffinity chromatography were assessed by SEC-HPLC, showing a > 99% purity (main peaks and high molecular weight species). The main peak profiles are virtually identical with molecular weights of 5.246 × 10^5^ Da for PF-D-Trimer and 5.223 × 10^5^ Da for PF-W-Trimer measured by MALS. Both molecular weights are consistent with trimers composed of three monomers of approximately 170 kDa. However, PF-W-Trimer exhibits slightly more high molecular weight species after purification (Fig. 1d). Data from electron microscopy confirm that immunoaffinity-purified PF-D-Trimer sample contains assembled spike trimers (Fig. 1e).

To investigate the stability of formulated PF-D-Trimer, samples were stored at 4 °C or 25 °C for varying times. The immunogenicity (Fig. 1f) of the stored samples was evaluated in Sprague-Dawley rats 28 days after a single dose immunization. The alum plus CpG 1018 formulated vaccine exhibits comparable immunogenicity across all temperatures and storage periods. The HEK293/hACE2 cell binding assay (Fig. 1g) is a functional assay that assesses the RBD ability to interact with the hACE2 surface receptor and, therefore PF-D-Trimer structural integrity. The assays demonstrate that the binding affinities of PF-D-Trimer dissociated from formulations stored at 4 °C and 25 °C for six months are almost identical. Taken together, the immunogenicity and the binding results suggest that formulations of PF-D-Trimer are stable when stored at 4 °C or 25 °C for up to six months.

### Induction of immune response by alum plus CpG 1018 adjuvanted PF-D-Trimer in mice

**Fig. 2:**
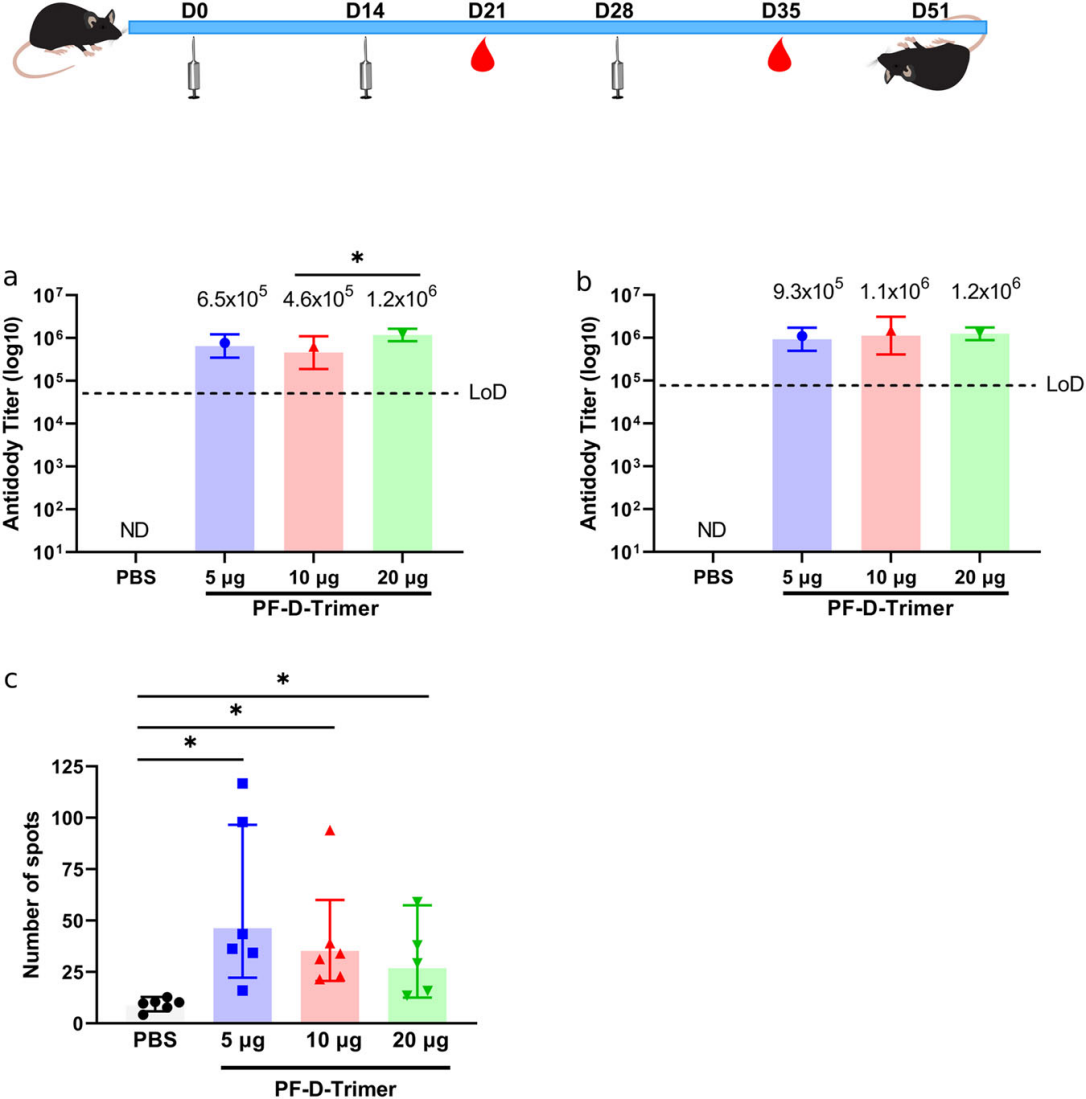
Immunogenicity of PF-D-Trimer in mice. C57BL/6 mice were immunized with three different doses of PF-D-Trimer formulated with alum plus CpG 1018, as indicated in the top panel and described in “Methods”. Sera were collected and the immune responses were analyzed one week after the second (Day 21) and the third (Day 35) injections. **a** PF-D-Trimer binding antibody ELISA titers at Day 21. **b** PF-D-Trimer binding antibody ELISA titers at Day 35. **c** IFN-γ^+^ phenotype by ELISpot after stimulation by S protein. Points represent individual animal; bars indicate geometric mean titers (GMT) responses (with geometric standard deviation) for antibody assays and geometric mean counts (GMC) responses (with geometric standard deviation) for ELISpot. *p ≤ 0.05, ***p ≤ 0.001, ****p ≤ 0.0001. ● 5 μg group, ▲10 μg group, ▼ 20 μg group. Dotted lines indicate the limits of detection, LOD; **a** 4 × 10^4^ **b** 8 × 10^4^. ND = the antibodies were not detected. Ordinary one-way ANOVA with Dunnett’s multiple comparison was used to analyze the differences among groups.

The immunogenicity of alum and CpG 1018 adjuvanted PF-D-Trimer as vaccine was first evaluated in C57BL/6 mice. Sera from these mice were evaluated for the amount of anti-spike IgG. Sera from the PBS formulation injected mice only showed titers at background level. At Day 21 (Fig. 2a), the antibody reciprocal GMT titers of the 5, 10, and 20 μg groups reached 6.5 × 10^5^, 4.6 × 10^5^, and 1.2 × 10^6^, respectively. The 10 μg and 20 μg groups displayed significant differences in antibody titers (*p* = 0.0329). At Day 35 (Fig. 2b), the antibody reciprocal GMT titers of the 5, 10, and 20 μg groups reached 9.3 × 10^5^, 1.1 × 10^6^, and 1.2 × 10^6^, respectively, and there was no significant difference in the antibody titers among the groups. Together these results demonstrate that the antibody responses were increased by the booster immunization.

Mice immune sera were further tested to identify whether the vaccine-adjuvant system could induce IFNγ^+^ splenocytes in the vaccinated mice of the immunization groups (Fig. 2c). Compared with the PBS control group, the 5, 10, and 20 μg groups had significant differences in the number of anti S protein specific IFNγ producing T cells. These results demonstrate that the formulated PF-D-Trimer is promoting the development and activation of IFNγ^+^ splenocytes cells in C57BL/6 mice.

### Immunogenicity of alum plus CpG 1018 adjuvanted PF-D-Trimer in Sprague-Dawley rats

**Fig. 3:**
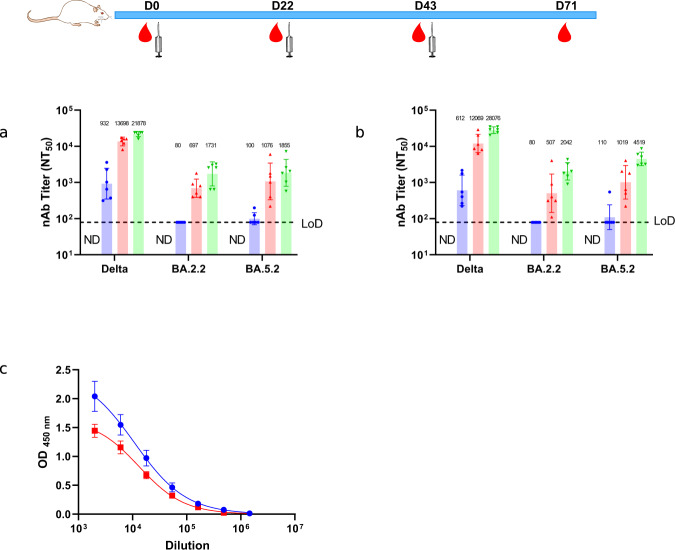
Induction of neutralization antibodies by PF-D-Trimer and IgG response toward the Trimer Domain in Sprague-Dawley rats. Sprague-Dawley rats were immunized with two different doses of PF-D-Trimer formulated with alum plus CpG 1018 as indicated in the top panel and as described in “Methods”. Sera were collected and the neutralization activities against Delta and Omicron BA.2.2 and BA5.2 were analyzed as described in Methods. **a** Sera neutralization activities with 10 μg of adjuvanted PF-D-Trimer. **b** Sera neutralization activities with 30 μg of adjuvanted PF-D-Trimer. Points represent individual animal; bars indicate geometric mean titers (GMT) responses (with geometric standard deviation). Every bar in different colour represents a different time point of blood collection ● Day 22, ▲Day 43, ▼ Day 71. Dotted lines indicate the limit of detection, LOD; 80. ND = At D0 the neutralizing antibodies were not detected. **c** ELISA binding curves of SD rats sera depleted with recombinant purified Monkeypox L1R-TD-Trimer to assess the removal of Trimer Domain-specific antibodies. Bars indicate standard deviation. ● Non-depleted sera, ■ Depleted sera.

We also examined the immunogenicity of alum and CpG 1018 adjuvanted PF-D-Trimer in Sprague-Dawley rats. During the progression of the COVID-19 pandemic many variants of SARS-CoV-2 emerged. Among them is Omicron, a highly mutated variant of concern highly resistant to vaccine-induced antibody neutralization^16^. Thereby, the immune sera were tested for their neutralization capabilities against the Delta and Omicron BA.2.2 and BA5.2 variants of SARS-CoV-2. The reciprocal ID_50_ GMT of 10 µg PF-D-Trimer adjuvanted with both alum and CpG 1018 reached 21878 for Delta, 1731 for Omicron BA.2.2 and 1855 for Omicron BA.5.2 after three doses at Day 71 (Fig. 3a). The reciprocal ID_50_ GMT of 30 µg adjuvanted PF-D-Trimer reached 28076 for Delta, 2042 for Omicron BA.2.2 and 4519 for Omicron BA.5.2 after three doses at Day 71 (Fig. 3b). Thus, neutralizing antibodies were generated against the three variants using the adjuvanted PF-D-Trimer, the highest neutralization being with Delta (homologous immunization), followed by Omicron BA.5.2 and then Omicron BA.2.2. Neutralization titers for Omicron BA.5.2 were higher than BA.2.2, regardless of the dose.

Results from the depletion ELISA demonstrate the induction of a humoral immune response directed toward the Trimer Domain but antibodies specific to the S protein ectodomain make a significant contribution to the overall ELISA signal (Fig. 3c).

### Induction of immune response by alum plus CpG 1018 adjuvanted PF-D-Trimer and PF-W-Trimer in Syrian golden hamsters

**Fig. 4:**
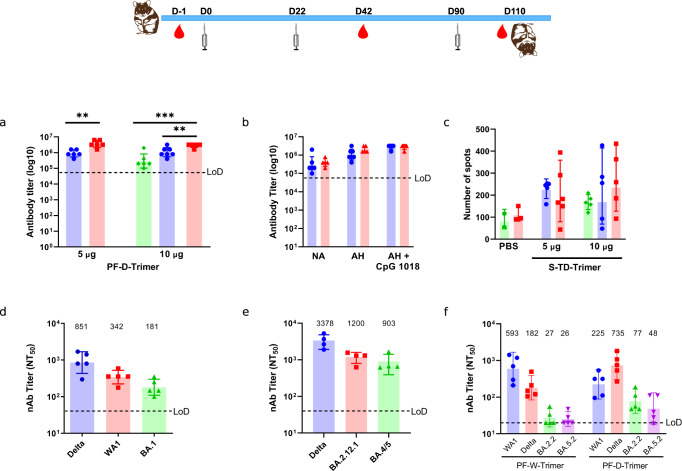
Immunogenicity of the PF-Trimers in Syrian golden hamsters. Syrian golden hamsters were immunized with two different doses of PF-Trimers formulated or not with alum plus CpG 1018, as indicated in the top panel and as described in Methods. Sera were collected and the immune responses were analyzed twenty days after the second and third injections (Day 42 and Day 110). **a** PF-D-Trimer binding antibody ELISA titers at Day 42. ♦ Not Adjuvanted, ● Alum, ■ Alum plus CpG 1018. **b** Comparison of different PF-D-Trimer (10 μg) formulations binding antibody ELISA titers at Day 42 and Day 110. NA, not adjuvanted; AH, alum; AH + CpG 1018, alum plus CpG 1018. ● Second immunization, ▲Third Immunization. **c** Detection of IFN-γ^+^ splenocytes by ELISpot after stimulation by PF-D-Trimer. ♦ Not Adjuvanted, ● Aluminium Hydroxide, ■ Alum plus CpG 1018. **d** Sera neutralization activities against WA1, Delta, and Omicron BA.1 from PF-D-Trimer immunized animals using a live virus neutralization assay. **e** Sera neutralization activities against SARS-CoV-2 Delta, BA.2.12.1 and BA.4/5 pseudotyped viruses from PF-D-Trimer immunized animals. **f** Comparative sera neutralization studies from PF-W-Trimer, and PF-D-Trimer immunized animals toward SARS-CoV-2 WA1, Delta, BA.2.2 and BA.5.2, using a live virus neutralization test. The titers below the limit of detection (LOD) were set to 20. ● WA1, ■ Delta, ▲BA.2.2, ▼BA.5.2. Points represent individual animal; bars indicate geometric mean titers (GMT) responses (with geometric standard deviation) for antibody assays and geometric mean counts (GMC) responses (with geometric standard deviation) for ELISpot. **p ≤ 0.01, ***p ≤ 0.001. Dotted lines indicate the limits of detection, LOD; **a** 5 × 10^4^. **b** 5 × 10^4^. **c** 5 × 10^4^. **d** 30. **e** 30. **f** 20. Two-tailed t-tests were used to compare the means of each two groups. Ordinary one-way ANOVA with Dunnett’s multiple comparison was used to analyze the differences among groups.

We evaluated the immunogenicity and potential efficacy of PF-D-Trimer and PF-W-Trimer formulated with or without alum and with or without CpG 1018 after two or three injections containing 5 μg or 10 μg of PF-D-Trimer in Syrian golden hamsters. Twenty days after the second injection (Fig. 4a), all the immunization groups, including the non-adjuvanted protein group were showing a humoral immune response. For PF-D-Trimer, the geometric means were 8.9 × 10^5^ and 3.6 × 10^6^ for the 5 μg groups adjuvanted with alum or alum plus CpG 1018 respectively, the later giving a significantly higher GMT titer. In the 10 μg groups the GMT were 1.1 × 10^6^ and 2.8 × 10^6^ for the formulations adjuvanted with alum or alum plus CpG 1018 respectively, the later being significantly higher. Interestingly, the unadjuvanted PF-D-Trimer was still giving a GMT of 2.9 × 10^5^ even though significantly lower than the adjuvanted formulations. The third injection boosted the GMT for the 5 μg (Supplementary Fig. 1) and the 10 μg groups (Fig. 4b), although these did not differ significantly from the second injection, regardless of whether the formulations were adjuvanted or not in each group.

Splenocytes from hamster groups harvested after immunization were tested for their IFNγ^+^ phenotype (Fig. 4c). There was no significant difference in the numbers of anti PF-D-Trimer-specific IFNγ producing T cells between the 5 and 10 µg groups irrespectively of the formulations. Moreover, the protein alone is also able to generate a T cell response. Taken together, these results suggest that PF-D-Trimer direct the hamsters’ T cells towards an IFNγ^+^ phenotype.

Sera from hamsters that had received three 5 μg injections of PF-D-Trimer formulated with alum plus CpG 1018 were used to assess their neutralization activities toward the ancestral WA1 and the Delta strains as well as Omicron variants of SARS-CoV-2 (Fig. 4d). The reciprocal ID_50_ GMT were 851 for Delta, 342 for WA1 and 181 for Omicron BA.1. Thus, neutralizing antibodies were generated against the three strains.

The sera were also used to assess their neutralization activities toward SARS-CoV-2 Delta, BA.2.12.1, and BA.4/5 pseudotyped viruses. The reciprocal ID_50_ GMT were 3378 for Delta, 1200 for BA.2.12.1, and 903 for BA.4/5 (Fig. 4e). Similar to the results of the experiments carried out with the real viruses, the results with the pseudotyped viruses also show a neutralization activity against the three strains.

In a separate experiment, we compared the neutralization activities of the sera from hamsters that had received two 5 μg injections of formulated PF-W-Trimer or PF-D-Trimer toward SARS-CoV-2 WA-1, Delta, BA.2.2 and BA.5.2, using a live virus neutralization assay (Fig. 4f). The results show higher neutralization titers toward viruses homologous to the S proteins used for immunization, WA1 or Delta. The neutralizing antibody titers from the sera of the animals immunized with PF-W-Trimer towards the Omicron BA.2.2 and BA.5.2 variants are lower than those of the immunizations with PF-D-Trimer. In the PF-W-Trimer immunization group, three out of five animals showed titers below the detection limit for the Omicron BA.2.2 and BA.5.2 variants. However, in the PF-D-Trimer immunization group, all hamsters have titers higher than the LOD for BA.2.2, whereas only one was below the detection limit for BA.5.2. These results support the appropriateness of using PF-D-Trimer as a vaccine antigen rather than PF-W-Trimer against the Omicron variants used in the neutralization assays.

### Immunogenicity and protective efficacy of adjuvanted PF-D-Trimer in K18-hACE2 H11 transgenic mice

The protective efficacy of PF-D-Trimer adjuvanted or not with alum plus CpG 1018 was examined in K18-hACE2 mice. hACE2 mice develop respiratory disease resembling severe COVID-19 and is a suitable model to study pathogenesis and immune responses^17^.

**Fig. 5:**
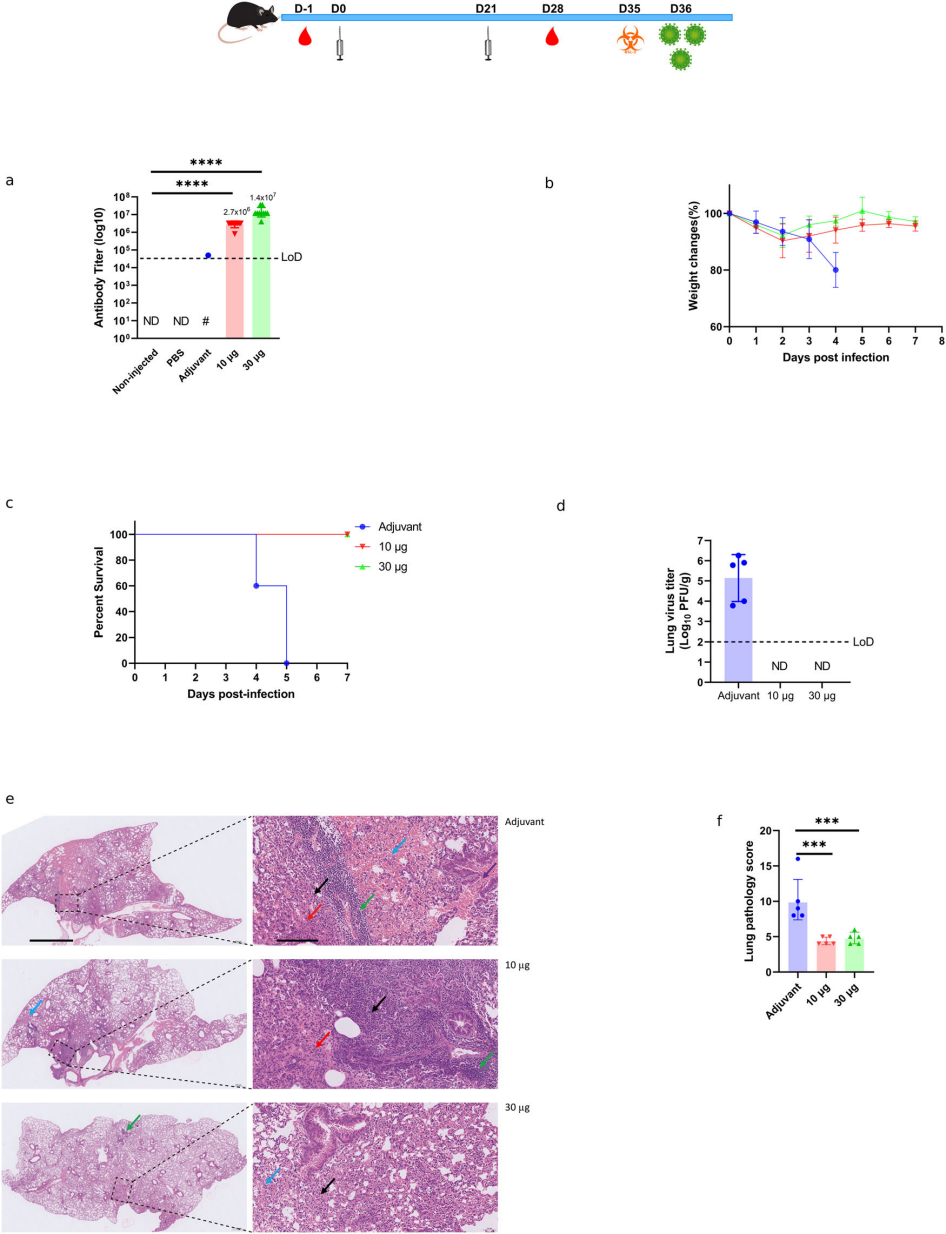
Immunogenicity and protective efficacy of PF-D-Trimer in K18-hACE2 H11transgenic mice. K18-hACE2 H11 mice were immunized with two different doses of PF-D-Trimer formulated with alum plus CpG 1018 or adjuvant only, as described in the top panel and in Methods. Sera were collected and the immune responses were analyzed one week after the second injection (Day 28). Two weeks after the second injection (Day 35), the mice were exposed to SARS-CoV-2 Delta variant at 1 × 10^5^ TCID_50_ per animal by the intranasal route. **a** PF-D-Trimer binding antibody ELISA titers at Day 28. **b** Weight change of mice after exposure to SARS-CoV-2 (points represent mean weight, bars indicate standard deviation). **c** Survival curves of mice inoculated with SARS-CoV-2. **d** Viral load analysis in lung tissues at necropsy (Day 3 post-challenge) in SARS-CoV-2 infected mice. Dotted lines reflect assay limit of detection (100 PFU/g). **e** Representative images of pathological analysis of mouse lungs on Day 3 post-challenge. The image on the right is a partial enlargement of the image on the left. The black arrow represents the thickening of the alveolar wall with inflammatory cell infiltration; the blue arrow represents alveolar haemorrhage; the purple arrow represents bronchial haemorrhage; the red arrow represents pulmonary edema, and tissue exudates can be seen in the alveolar space. Left images: scale bar 1 mm. Right images: scale bar 0,1 mm. **f** Lung histopathology scores 3 days post infection. The mice were euthanized and lung tissue samples were collected for sectioning and staining. The histopathology sections were scored as outlined in the methods. **a**, **d**, **f** Points represent individual animal; bars indicate geometric mean titers (GMT) responses (with geometric standard deviation). ● Adjuvant only, ▼PF-D-Trimer 10 μg and adjuvant, ▲PF-D-Trimer 30μg and adjuvant. ***p ≤ 0.001, ****p ≤ 0.0001. Dotted lines indicate the limits of detection, LOD; **a** 4 × 10^4^ **d** 1 × 10^2^ PFU/g. ND = not detected. # not detected in 9 out of 10 mice. One-way ANOVA with Dunnett’s multiple comparison was used to analyze the differences among groups.

We examined the immunogenicity of alum plus CpG 1018 adjuvanted PF-D-Trimer in K18-hACE2 mice. The immune sera collected after two immunizations were evaluated for the amount of anti-spike IgG (Fig. 5a). The antibody reciprocal GMT of the 30 μg group reached 1.4 × 10^7^ while for the 10 μg group, although the titer was lower compared to the 30 μg group, it was still significantly higher than the control groups with a titer of 2.7 × 10^6^. Mice vaccinated with only the adjuvant and intranasally infected with the Delta strain (Fig. 5b) were showing a decrease in weight and died at 4 or 5 DPI (Fig. 5c). For the mice vaccinated with 10 μg or 30 μg of adjuvanted PF-D-Trimer, the body weight slightly decreased in the first 2 days after infection, and subsequently recovered. The mice in the PF-D-Trimer vaccinated groups were in good condition at 7 DPI and all survived. The viral load in the mice’s lung tissue was detected on the third day after infection (Fig. 5d). The live virus titer in lung tissue was below the minimum detection limit (100 PFU/g) in the mice from the 10 and 30 μg of adjuvanted PF-D-Trimer groups, while load from the mice of the adjuvant only group reached more than 1 × 10^5^ PFU/g of lung tissue.

The results of the lung pathological analysis at D3 after infection (Fig. 5e, f) were in agreement with the viral loads; the bronchial and alveolar structures of the lung tissue of the mice in the 10 and 30 μg adjuvanted PF-D-Trimer groups were intact and clear. There was some local alveolar wall thickening, occasionally accompanied by a small number of inflammatory cells, lymphocyte infiltration and a small amount of angioedema, but the overall lesions were mild or moderate. However, tissue from mice of the adjuvant only group showed severe pathological features; there were large areas of alveolar wall thickening, accompanied by more inflammatory cell infiltration, more pulmonary edema and local haemorrhage at the tissue edge. Mice in the adjuvant-only group had significantly higher lung pathology scores compared with the scores of mice in the adjuvanted PF-D-Trimer 10 and 30 μg groups.

## Discussion

The PF-Trimers vaccine candidates come in the form of the ectodomain of the S protein of SARS-CoV-2 fused to TD, our proprietary trimerization domain. The PF-Trimers are in a disulfide bond-linked homotrimeric form, which permit the acquisition of important epitopes only found in the native trimeric form of the S proteins of coronaviruses and are so trimer restricted^7^. Additionally, the trimeric form of the SARS-CoV-2 S protein adopts an open-trimer conformation potentially allowing the development of broadly protective or pan-coronavirus vaccines by exposing highly conserved region of the protein^8^.

The PF-Trimers are expressed at high levels in CHO cells under bioreactor culture conditions commonly found in the biopharmaceutical industry and do not require specialized equipment beyond what is readily available from recognized manufacturers in the industry. Our expression level of approximately 500 mg/L in a bioreactor is in the upper range compared to other previously reported trimerized S protein constructs (100 to 500 mg/L of cell supernatant)^18–20^. The presence of the TD domain and the α−TD domain monoclonal antibody allows an easy one-step immunoaffinity purification of the protein, which coupled with a high expression titer, permit the implementation of a commercially favourable downstream processing, manufacturability, and production.

In many developing countries, limitations of existing cold chains provide challenges for vaccine distribution, therefore, only a tiny fraction of the vaccines against COVID-19 have been administered there. We demonstrate that formulated PF-D-Trimer remains stable when stored at 4 °C or 25 °C for at least six months, as evaluated by immunization studies in rats and cell-binding results. Thus, it is likely that its distribution might be possible without refrigeration, which increases its global accessibility.

The immunogenicity studies have shown that PF-D-Trimer adjuvanted with CpG 1018 plus alum was effective in inducing a potent immune response in four animal models: C57BL/6 mice, Sprague-Dawley rats, Syrian golden hamsters, and K18-hACE2 mice, the latter being completely protected in a challenge with a live virus.

In C57BL/6 mice, vaccination with PF-D-Trimer adjuvanted with CpG 1018 and alum elicited a robust humoral immune response regardless of the dose used for immunization. We have also confirmed that a booster dose increases the antibody titer, which is very relevant since homologous and heterologous booster doses are now an integral part of anti-COVID-19 vaccine strategies^21,22^. In addition to the humoral immune response, type 1 S-specific T-cell biased immunity likely contributes to the protection of COVID-19 convalescents^23^. Our in vitro T cell analyzes demonstrated that PF-D-Trimer vaccination adjuvanted with CpG 1018 and alum elicited robust anti-recombinant SARS-CoV-2 S protein trimer specific T cells that may contribute to protection against SARS-CoV-2. These results are consistent with the high correlation seen between humoral and cellular immune responses to SARS-CoV-2^24^.

The neutralization studies have shown that SD rats immunized with PF-D-Trimer adjuvanted with alum plus CpG 1018 develop robust neutralizing antibodies against the Delta, Omicron BA.2.2, and BA.5.2 variants. We observe a reduction for the Omicron variants compare to Delta. However, the reduction is less pronounced for BA.5.2 than for BA.2.2. Results from the depletion ELISA have shown that Trimer Domain is immunogenic and, therefore, part of the immune response is directed towards it. But the response directed towards the S protein ectodomain is significant as demonstrated by the IgG and neutralization titers throughout this study. A humoral immune response directed towards a heterologous trimerization domain has previously been reported^25^.

In our immunogenicity studies of the PF-Trimers in the hamster model, we assessed the impact of the formulation on general antibody titers. Similar to the results observed in mice, the formulation combining PF-D-Trimer with alum plus CpG 1018 appears to generate higher titers. We have also observed that this formulation directs the immune response towards a cellular immune response. The hamsters’ neutralization studies have also shown that the animals immunized with the same formulation develop neutralizing antibodies directed against WA1, Delta, Omicron BA.1, BA2.12.1, and BA.4/5. The PF-W-Trimer and PF-D-Trimer comparative neutralization studies demonstrate that PF-D-Trimer induces higher neutralization titers than the prototype WA1 Spike protein trimer against the BA.2.2 and BA.5.2 Omicron variants, making it a more appropriate choice of vaccine antigen against these variants.

We have also demonstrated that PF-D-Trimer adjuvanted with CpG 1018 and alum induces a protective immunity in K18-hACE2 transgenic mice at the doses used for immunization. Although vaccinated mice lose weight early in the infection, they quickly return to their original weight, whereas unvaccinated mice continue to lose weight until they die. Among the vaccinated mice, the viral loads were undetectable and the lungs lesions were mild or moderate, whereas the load of the control group was very high with severe pathological features. Published research results have indicated that high viral loads are associated with mortality for symptomatic and hospitalized patients who tested positive for SARS-CoV-2^26^.

New SARS-CoV-2 variants are emerging as the virus evolves and escapes from antibody selection pressure. In addition, recombination is extremely common in the evolutionary history of SARS-like coronaviruses and is expected to shape SARS-CoV-2 in the coming years^27^. As the Delta variant began to progressively disappear near end of 2021, a new variant, Omicron BA.1 appeared and spread very quickly. SARS-CoV-2 Delta and Omicron variants have co-circulated in the United States, allowing co-infections and possible recombination events^10^. Studies have revealed that Omicron BA.1 was less likely to be neutralized by convalescent plasma from COVID-19 patients or plasma from people who received vaccines based on WA1^13,28,29^. Our neutralization results present a differentiating factor since PF-D-Trimer is based on the Delta strain while most of the published studies use vaccines based on WA1. We do observe a 4.7 reduction for the hamster model in the neutralization titer for Omicron BA.1; however, this seems to be located in the lower region of the range obtained with the WA1-based vaccines, which varies between 6 and 23 times.

The spike protein consists of two subunits, S1 and S2. S1 mediates binding to the receptor by the interaction of the receptor binding domain (RBD) with ACE2. Studies have indicated that most potent neutralizing antibodies target RBD to block the interaction with ACE2^30–32^. The Delta variant shares a common mutation, T478K, with BA.1 in RBD, which could partly explain the lower reduction of neutralizing antibodies when using PF-D-Trimer instead of the other vaccines based on the original WA1 spike protein. A reduction in the neutralization titers was also observed in our hamster model, but to a lesser extent, towards the original WA1 strain. The reduction can be explained by the T478K mutation but also by L452R, the latter being known to be an escape mutation for Bamlanivimab^33^.

SARS-CoV-2 is in constant evolution. BA.2.12.1 evolved from BA.2 in the United State in the spring of 2022 while Omicron BA.4 and BA.5 were identified in South Africa in January and February 2022. These variants are outcompeting the previously circulating BA.1 and BA.2 variants. In the summer of 2022, BA.5 was responsible for almost 90% of COVID-19 cases in the United States. Meanwhile, BA.4 was responsible for about 5% of cases^34^. The spike protein of BA.2.12.1 contains the L452Q and S704L mutations in addition to the known mutations in BA.2. The spike proteins of BA.4 and BA.5 are identical, each with the additional mutations Δ69–70, L452R, F486V. Studies^35,36^, identified that the specific mutations found in BA.2.12.1 and BA.4/5 contribute to antibody resistance, BA.4/5 showing a more pronounced reduced neutralization than BA.2.12.1 to the vaccination induced by the Pfizer–BioNTech BNT162b2 vaccine. Hachmann, N.P. et al.^37^. have shown that the BA.2.12.1, BA.4, and BA.5 subvariants substantially escape neutralizing antibodies in sera from participants who had been infected with BA.1 or BA.2.

As the newly emerged variants represent a significant proportion of new cases of infection, it becomes imperative to develop a vaccine that can generate adequate protection against them. Interestingly, Qu, P. et al.^13^ demonstrates that sera from unvaccinated or vaccinated Delta-wave ICU patients more strongly neutralize BA.4/5 and BA.2.12.1 compared to BA.1 and BA.2, while infection during the BA.1 wave did not appear to offer effective protection against the new sublineages. We also observe higher neutralization titers against BA.5.2 compared to BA.2.2 with sera from SD rats immunized with PF-D-Trimer. It is worth noting that the BA.4/5 sublineages and Delta share the immune escape associated L452R mutation^38^, which could explain why PF-D-Trimer can induce high neutralization titers against BA.5.2.

It is known that neutralization antibody levels are highly predictive of immune protection^39^. Our results are showing that adjuvanted PF-D-Trimer induces high neutralization titers for different SARS-CoV-2 variants, including Omicron BA.2.2 and BA.5.2. In sum, the Delta spike protein could represent an effective booster vaccine antigen by activating cross-neutralizing B cells and a T cell response.

Delta infection was found to be broadly immunogenic in mice. Sera from likely Delta breakthrough cases can also neutralize VLPs generated with S genes from the Delta and BA.1 strains^40^. In SARS-CoV-2 pseudotyped virus neutralization assays, a Delta breakthrough infection (BTI) in double vaccinated subjects induces more potent cross-neutralizing antibodies against variants including BA.1 than Delta infection in unvaccinated subjects. As such a Delta BTI was determined to be an effective booster that could provide broad protection^41^. In vaccinated Delta-infected patients, RBD IgG titers are superior compared to BA.1-infected subjects, and the CD8 + T cells are more spike oriented^42^.

PF-D-Trimer consists of the full Delta S-protein ectodomain, including the S1 and S2 subdomains, trimerized by the Trimer Domain. Following immunization, the immune response appears to target more variable and likely more immunogenic epitopes in the RBD and N-terminal domain of S1 of SARS-CoV-2 strains^43,44^. The S2 subunit, which is more sequence conserved than S1, harbours a significant proportion of antibody-targeted epitopes in the response to SARS-CoV-2 infection or vaccination. Thus, cross-reacting antibodies targeting conserved regions in SARS-CoV-2 S2, such as the fusion peptide, heptad repeats, stem helix, or membrane proximal regions, are also stimulated by SARS-CoV-2 infection or vaccination^45,46^. One such antibody is HCLC-031 that efficiently neutralizes Delta and Omicron BA.1^47^. The oligomerization process modulates the antigenicity of the coronavirus S protein, in addition to the epitopes located on the monomeric form, some are trimer restricted^7^. Also, the trimeric form of SARS-CoV-2 S adopts an open conformation that exposes the S2 interface of the trimer and its highly conserved epitopes^8^. It is thus likely that PF-D-Trimer is a more complete immunogen than vaccines based on domains or subdomains of the S protein.

Our data demonstrate that PF-D-Trimer adjuvanted with alum plus CpG 1018 is stable up to six months without refrigeration, can induce cross-reactive humoral and cellular immune responses in various animal species and protective immunity against a homologous SARS-CoV-2 challenge in K18-hACE2 H11 transgenic mice. The adjuvanted PF-D-Trimer vaccine candidate has already passed the necessary safety assessments for advancement to human clinical trials as well as Investigator Initiated Trials (IITs) to define the clinical objectives. Together, the results support the advancement of adjuvanted PF-D-Trimer into human clinical studies to further demonstrate safety, immunogenicity, and vaccine efficacy. The advantage brought by the Trimer Domain for the stabilization of the antigen, the streamlined downstream processing, and CMC makes it possible to envisage a rapid scale-up of the production and rapid antigen interchangeability during the inevitable evolution of SARS-CoV-2.

## Methods

### Animals and biosafety experiments

All the animals’ experiments, except the experiments with the K18-hACE2 H1 mice, were approved by the Hubei University of Technology Laboratory Animal Ethics Review Committee (Approval number: 2021018). The K18-hACE2 H1 mice viral infections were conducted in the biosafety level 3 (BSL-3) facility at Wuhan Institute of Virology under a protocol approved by the Laboratory Animal Ethics Committee of Wuhan Institute of Virology, Chinese Academy of Sciences (Permit number: WIVA26202201). All works with live SARS-CoV-2 virus titration and neutralization assays were performed inside biosafety cabinets in the biosafety level 3 (BSL-3) facility at Hubei Provincial Centre for Disease Control and Prevention. Specific pathogen-free (SPF) female C57BL/6 mice (6–8 weeks old), SPF female Syrian hamsters (5–6 weeks old) and 6-week-old Sprague–Dawley rats were purchased from the Hubei Laboratory Research Centre. H11-K18-hACE2 male mice (6–8 weeks) were purchased from Jiangsu Jicui Yaokang Biotechnology Co., Ltd. Animals had free access to water and food in a controlled environment with a 12 h light/dark cycle (temperature: 16–26 °C, humidity: 40–70%).

### Protein trimerization, expression, and purification

The SARS-CoV-2 S glycoproteins B.1.617.2 (PF-D-Trimer) and WA1 (PF-W-Trimer) sequences were downloaded from GISAID and GenBank (accession numbers EPI_ISL_1970349 and QHR84449 respectively). The S genes were codon optimized for high level expression in CHO mammalian cells and biochemically synthesized by Genscript (China). Compared to the reference gene, the following modifications have been made during the synthesis: three mutations (RRAR to GSAS) were introduced in the S1/S2 furin site of the full-length SARS-CoV-2 S protein along with the 2P mutation; the transmembrane and the cytoplasmic domains were deleted; and the trimerization domain (TD) was added at the C-terminus. The recombinant SARS-CoV-2 S PF-D-Trimer gene was cloned in the pGenHT1.0-DGV plasmid with a 5’CMV promoter and a 3’ polyA sequence while the PF-W-Trimer gene was cloned in pEE12.4. The gene sequences of the recombinant SARS-CoV-2 S proteins with GSAS, 2P, and TD were confirmed by DNA sequencing. The linearized target plasmids were transfected into CHOK1-GenS or suspension culture-adapted CHOK1 cells to generate the stable cell pools. Subsequent screening for high-titer production clones, process optimization, and a fed-batch serum-free cell culture process in bioreactor, led to the production of recombinant PF-Trimers as highly secreted proteins in the culture medium of CHO cells.

The α−TD monoclonal antibody was produced in Sf9 insect cells by using a Bac-to-Bac recombinant baculovirus system (Invitrogen). The monoclonal antibody manufacturing process started with the revival, expansion, and production of the Sf9 cells from the working cell bank (WCB) into shake flasks and bioreactor using serum-free medium (Vbiosci). The cultured cells were infected by inoculation of a recombinant baculovirus carrying the α−TD monoclonal antibody genes. At the end of the infection phase, the cell culture supernatant with the secreted antibody was harvested by centrifugation. The clarified medium was subjected to a purification process by MabSelect PrismA (Cytiva) affinity chromatography according to the manufacturer’s instruction. The affinity chromatography eluate was subjected to concentration and diafiltration using TFF (Sartorius Stedim Biotech GmbH). In order to produce the immunoaffinity resin for PF-D-Trimer purification, the α-TD monoclonal antibody was coupled to NHS-activated Sepharose 4 Fast Flow (Cytiva) according to the manufacturer’s instructions.

The PF-Trimers active substance manufacturing process started with the revival, expansion, and production of the CHO cells from the working cell bank (WCB) into shake flasks and bioreactor (Applikon) using serum-free medium (Gibco). At the end of the growth phase, the cell culture supernatants were harvested by depth filtration (Sartorius Stedim Biotech GmbH). The clarified media were subjected to a purification process by α−TD monoclonal antibody immunoaffinity chromatography to capture the PF-Trimers, and virus inactivation by a low pH treatment. The immunoaffinity chromatography eluates were subjected to concentration and diafiltration using tangential flow filtration (TFF) (Sartorius Stedim Biotech GmbH) and final 20 nm nanofiltration (Pall Corporation) to obtain the purified SARS-CoV-2 recombinant PF-Trimers active substances. At the end of the purification process, endotoxins were measured by a TAL assay (Zhanjiang A&C Biological Ltd) and were within the acceptable levels as defined by the Chinese Pharmacopoeia.

### PF-Trimers purity, molecular weight, aggregation and fragmentation analysis by SEC-HPLC and MALS-HPLC

The α-TD immunoaffinity purified Trimers were analyzed by Size-Exclusion Chromatography (SEC-HPLC) using a Shimadzu LC-2030 HPLC (Shimadzu Corporation, Japan) with an analytical SRT SEC-300 7.8 x 300mm column (Sepax). Phosphate-buffered saline (PBS) was used as the mobile phase with OD_280_ nm detection over a 20 min period at a flow rate of 1 ml/min. The samples were analyzed by multi-angle static light scattering (DAWN, Wyatt Technology) to measure their molecular weights.

### Negative stain electronic microscopy

To characterize the oligomerization status of immunoaffinity purified PF-D-Trimer, 20 μl of samples was added dropwise to 200-mesh grids (Fanghua Film Copper Mesh) and incubated at room temperature for 10 min. Then the grids were negatively stained with 2% phosphotungstic acid for 3 min, and the remaining liquid was removed with a filter paper. The prepared samples were observed with a HT7800 (Hitachi) transmission electron microscope.

### Immunogenicity of the PF-Trimers

Twenty-eight C57BL/6 mice were randomly divided into 4 immunization groups; PBS with 50 μg aluminium hydroxide (hereafter abbreviated as alum) plus 10 μg CpG 1018 (General Biol), 5 μg PF-D-Trimer with 50 μg alum plus 10 μg CpG 1018, 10 μg PF-D-Trimer with 50 μg alum plus 10 μg CpG 1018, 20 μg PF-D-Trimer with 50 μg alum plus 10 μg CpG 1018. The total injection volume of the formulated vaccines was 100 µL per dose. Given the volumes used for the injections and for welfare considerations to the experimental animals^48^, three doses were administered by multipoint subcutaneous injection, at Day 0, Day 14, and Day 28. The sera of immunized mice were collected by retro-orbital bleeding at Day 21 and Day 35 to detect the SARS-CoV-2-specific IgG endpoint GMT. Spleens were removed for ELISpot assays after sacrifice by cervical dislocation at Day 51.

Sprague-Dawley rats were immunized intramuscularly (IM) with two different doses of PF-D-Trimer adjuvanted with 375 µg alum plus 0.75 mg CpG 1018: five rats with 10 µg of protein, and six rats with 30 µg of protein. The total injection volume of the formulated vaccines was 500 µL per IM dose. Three IM doses were administered (at Day 0, Day 22, and Day 43). The sera were collected at Day 22, Day 43, and Day 71 to detect SARS-CoV-2 specific neutralizing antibodies as described below. For the PF-D-Trimer stability assays, the rats were injected intramuscularly with 10 µg PF-D-Trimer formulated with alum plus CpG 1018 that had been stored at 4 °C or 25 °C for up to five months. The sera were collected 28 days after the immunization.

Syrian hamsters were divided into 7 groups (PBS alone, PBS with 75 μg alum plus 150 μg CpG 1018, 10 μg of PF-D-Trimer alone, 5 μg PF-D-Trimer only with 75 μg alum, 10 μg PF-D-Trimer only with 75 μg alum, 5 μg PF-D-Trimer with 75 μg alum plus 150 μg CpG 1018, 10 μg PF-D-Trimer with 75 μg alum plus 150 μg CpG 1018). The total injection volume of the formulated vaccines was 300 µL per intra-muscular (IM) dose. Three IM injections were administered (at Day 0, Day 22, and Day 90). The sera were collected at Day 42 and Day 110 to detect the SARS-CoV-2-specific IgG endpoint GMTs and neutralizing antibodies as described below. Spleens were removed for ELISpot assays after sacrifice at Day 110. For the comparative PF-W-Trimer and PF-D-Trimer neutralization studies, the animals were immunized with 5 μg of protein adjuvanted with 75 μg alum plus 150 μg CpG 1018. Two IM injections were administered (at Day 0 and Day 22). The sera were collected at Day 42 to detect the neutralizing antibodies as described below.

### SARS-CoV-2-specific antibody endpoint GMT measurement with ELISA

Specific IgG antibody endpoint GMT were measured by ELISA. Briefly, sera serially diluted in ELISA buffer (1% BSA with 0,05% Tween-20) were added (100 μl/well) to 96-well plates (Costar) coated with recombinant SARS-CoV-2 S protein antigen (Novozan Biotechnology Co., Ltd.) and blocked with ELISA buffer for 120 min at 37 °C. After three washes with wash buffer (PBS with 0.05% Tween-20), the plates were incubated at 37 °C for 30 min with horseradish peroxidase-conjugated goat anti-mouse IgG (Proteintech, SA00001-1) or goat anti-hamster IgG (Shanghai Universal Biotech Co., Ltd., 127-035-160) diluted in ELISA buffer at final concentrations of 0.08 μg/μl. The plates were then washed 3 times with wash buffer. Signals were developed using TMB substrate (Solarbio). The colorimetric reaction was stopped by the addition of 2 M H_2_SO_4_. Finally, the absorbance (450 nm) was measured with a Varioskan LUX Multimode Microplate Reader (Thermo). The IgG endpoint GMT were defined as the dilution fold with an optical density exceeding the average background plus 3 times the standard deviation (sera from the PBS control groups). The Limit of Detection (LoD) was defined as the reciprocal of the highest concentration of sera tested.

### Immune response directed toward the Trimer Domain by depletion ELISA

For the depletion ELISA, 10 µg/mL of an unrelated trimerized depletion antigen (Monkeypox L1R trimerized with the Trimer Domain, L1R-TD-Trimer) was added to the sera of Sprague-Dawley rats immunized with PF-D-Trimer. The sera were incubated at 37 °C for 1 h before being added to the ELISA plate coated with PF-D-Trimer and analyzed as described above. The ELISA plate configuration included separate rows coated with the depletion antigen as a control for incomplete depletion of Trimer Domain-specific responses.

### ELISpot assays

Spleens from immunized animals were removed at Day 51 for the mice and at Day 110 for the hamsters and splenocytes were isolated for protein S-specific T-cell detection with the ELISpotPLUS mouse IFN-γ or ELISpotPLUS hamster IFN-γ kits (Mabtech) according to the manufacturer’s instructions. Briefly, 3–5 × 10^5^ splenocytes/well and 4 μg/well SARS-CoV-2 spike protein B.1.617.2 (Nanjing Vazyme Biotech Co., Ltd) were mixed and incubated at 37 °C. After 48 h followed by 5 washes with PBS, the probe antibodies (anti mouse IFN-γ, Mabtech, 3321-4APW-2; anti hamster IFN-γ, Mabtech, 3102-4APW-2) were added at a concentration of 1 μg/μl and incubated at room temperature for 2 h. After washing 5 times with PBS, alkaline phosphatase labelled streptavidin was added at a dilution of 1 in 1000 and incubated for 1 h at room temperature. After washing 5 times with PBS, alkaline phosphatase labelled streptavidin was added at a dilution of 1 in 1000 and incubated for 1 h at room temperature. After washing 5 times with PBS, the chromogenic solution (BCIP/NBT-plus) was added and incubated at room temperature for 10 min. Colour development was then stopped with deionized water. The number of dots in the wells of the ELISpot plate was analyzed using an ELISpot reader system (AID ELISpot Reader Classic and AID ELISpot Reader Software, Autoimmun Diagnostika GmbH).

### Pseudovirus-based neutralization assay

The 50% neutralization titer (NT50) was measured using a modification of the procedure from Nie, J. et al.^49^. Briefly, the sera from immunized animals were diluted 3 times, starting from 1:33.33, and incubated with SARS-CoV-2 pseudovirus (2 × 10^4^ TCID_50_/mL) at 37 °C for 60 min. DMEM without serum was used as a negative control group. Then the HEK293T-hACE2 cells were added to each well (2 × 10^4^ cells/well) and incubated at 37 °C for 48 h. Luciferase activity, which reflects the degree of SARS-CoV-2 pseudovirus transduction, was measured using Bio-Lite Luciferase Assay System (Vazyme). The NT50, calculated by the Reed-Muench method^50^, was defined as the fold dilution, which obtained more than 50% inhibition of pseudovirus transduction compared to the control group. The Limit of Detection (LoD) was defined as the reciprocal of the highest concentration of sera tested.

### SARS-CoV-2 neutralization assays

Vero E6 cells (2.5 × 10^4^ cells/well) were seeded in 96-well plates and incubated overnight. Sera were inactivated at 56 °C for 30 min and diluted in serum-free DMEM at an initial dilution factor of 8, and then further serially diluted. The diluted sera were then mixed in a 1:1 ratio with the SARS-CoV-2 viruses (Hubei Provincial Centre for Disease Control and Prevention, WA1-Hu-1, Delta YJ20210701-01, Omicron BA.1 249099, Omicron BA.2.2 YJ20220413-11 and Omicron BA.5.2 YJ20220704-03 at 100 TCID_50_/100 μl and incubated 1 h at 37 °C. Then, the diluted sera/virus mixtures were added to the Vero cells and incubated at 37 °C with 5% CO_2_ for four days. The cells were monitored for cytopathic effect (CPE) every 24 h under an inverted microscope for each sera dilution. The neutralization end point 50%, the dilution of serum that can protect 50% of cells from CPE, was calculated by the Reed-Muench method^50^ to obtain the neutralizing antibody titer of each serum. The Limit of Detection (LoD) was defined as the reciprocal of the highest concentration of sera tested.

### HEK293/hACE2 stable cell line binding assays

PF-D-Trimer was dissociated from the formulations by incubation in a potassium phosphate buffer (K_2_HPO_4_:KH_2_PO_4_ at a 21:4 ratio, total PO_4_ ions concentration 1 M) at a 1:1 ratio for 2 h at room temperature. After centrifugation at 17000 x g for 15 min, the supernatants were concentrated and filtered on 0.22 μm.

For the cell-binding assays, 5 × 10^4^ HEK293/hACE2 cells were incubated at 4 °C for two hours with varying concentrations of dissociated PF-D-Trimer. After the incubation, the cells were spun down at 800 × *g* for 5 min at 4 °C and washed with 1 mL of cold PBS three times in total. The cells were then incubated with a 10 µg/mL dilution of iFluor 405 labelled anti-TD antibody for 1 h at 4 °C. After the incubation, the cells were spun down at 800 × *g* for 5 min at 4 °C and washed with 1 mL of cold PBS three times in total. The labelled cells were analyzed with a NovoCyte 3000 Flow Cytometer (Agilent). The mean fluorescence intensities were fitted in GraphPad Prism using a specific binding with Hill slope equation.

### K18-hACE2 H11 mice immunization and challenge study

K18-hACE2 H11 (C57BL/6JGpt) male transgenic mice were divided in 5 vaccination groups (#1 not immunized, 6 animals; #2 PBS control group, 7 animals; #3 PBS with 125 µg alum plus 750 µg CpG, 10 animals; #4 10 µg PF-D-Trimer with 125 µg alum plus 750 µg CpG, 10 animals; #5 30 µg PF-D-Trimer with 125 µg alum plus 750 µg CpG, 10 animals). Blood was collected before the immunization as a pre-immune control. Two subcutaneous 500 μl (5 dorsal multipoint injections of 100 μl) doses were administered (at Day 0 and Day 21). The sera were collected at Day 28 to detect the SARS-CoV-2-specific IgG endpoint GMT and neutralizing antibodies as described. At Day 35, mice from groups #3 to #5 were transferred to a BSL3 laboratory (Wuhan Institute of Virology). For the challenge study, the mice were intranasally infected with SARS-CoV-2 Delta (CRST: 1633.06.IVCAS 6.7593) at 1 × 10^5^ plaque-forming unit (PFU) (50 μl per animal). Change in body weight and determination of survival rate were monitored daily. Half of the animals were euthanized at 3 days post infection (dpi) and the remaining animals in each group were followed until day 7. Lung tissue samples from the euthanized mice were collected to determine the virus titer by plaque assay or fixed with 4% paraformaldehyde and stained with hematoxylin and eosin for pathological analysis. The lung sections were evaluated for alveolar wall thickening, inflammatory cell infiltration, pulmonary edema, bleeding and perivascular cuffing according to the scoring system described in Table 1. Quantification of viral titer in lung tissue by cell culture infectious assay (plaque assay) was performed as follows. Vero E6 cells were plated into 24-well plates 1 day in advance at a density of 10^5^ cells/well. Lung homogenates were prepared from 100 mg of lung tissue in 1 ml PBS. The next day, the cells were infected by tenfold serial dilutions of 100 μl lung homogenate supernatant for each sample and incubated at 37 °C for 1 h. The virus dilution was then removed and 1% methylcellulose was added. The plates were incubated for 4 days at 37 °C. The upper layer in the wells was discarded and 1 ml of fixative staining solution (3.7% formaldehyde + 1% crystal violet) was added. The cells were treated overnight at room temperature. After the fixative staining solution was rinsed with running water and dried, the number of viral plaques was counted. The plaque forming units per mL (PFU/ml) were determined using the following formula: (# plaques × dilution factor)/0.1 ml. An absence of plaque was scored as <1 and used to calculate the lower limit of detection. The PFU/ml values were adjusted for a volume of 1 ml and 1 g of tissue to calculate the plaque forming units per gram of tissue.

**Table 1:** Lung histopathology scoring system.

Score	Type	Observation
0	Within the normal range	Under the study conditions, the tissue is considered normal, taking into account factors such as the age and sex of the animal. Variations that occur under other conditions can be considered abnormal.
1	Very slight	The change is just outside the normal range.
2	Slight	Lesions can be observed, but not serious.
3	Medium	The lesions are obvious and likely to be more severe.
4	Serious	The lesions are very severe (the lesions have taken up the entire tissue and organ).

### Statistical analysis

Data were arranged in Excel and analyzed using GraphPad Prism 8.0.1 or 8.0.2. Two-tailed t-tests were used to compare the means of each two PF-D-Trimer immunized groups. Ordinary one-way ANOVA with Dunnett’s multiple comparison was used to analyze the differences among groups.

### Reporting summary

Further information on research design is available in the Nature Research Reporting Summary linked to this article.

## Supplementary information

Supplementary information

REPORTING SUMMARY

## Data Availability

Correspondence and requests for materials should be addressed to Yan Xu.
