# Supplementary material for: PF-D-Trimer, a protective SARS-CoV-2 subunit vaccine: immunogenicity and application
Source: NPJ Vaccines. 2023 Mar 15;8:38. doi: 10.1038/s41541-023-00636-8 (PMC10015519; doi:10.1038/s41541-023-00636-8)
Supplement: Supplementary file 1 — Supplementary information [file 41541_2023_636_MOESM1_ESM.pdf]

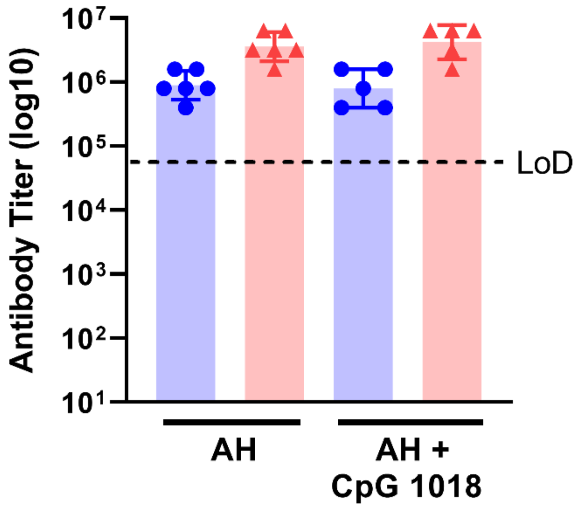

Supplementary Figure 1. Immunogenicity of PF-D-Trimer in Syrian golden hamsters. Syrian golden hamsters were immunized with PF-D-Trimer (5 µg) formulated or not with alum plus CpG 1018, as described in Material and Methods. Sera were collected and the immune responses were analyzed twenty days after the second and third injections (Day 42 and Day 110). AH, alum; AH + CpG 1018. ● Second immunization, ▲Third Immunization. Points represent individual animal; bars indicate geometric mean titers (GMT) responses (with geometric standard deviation). Dotted line indicates the limit of detection, LOD; $5x10^4$

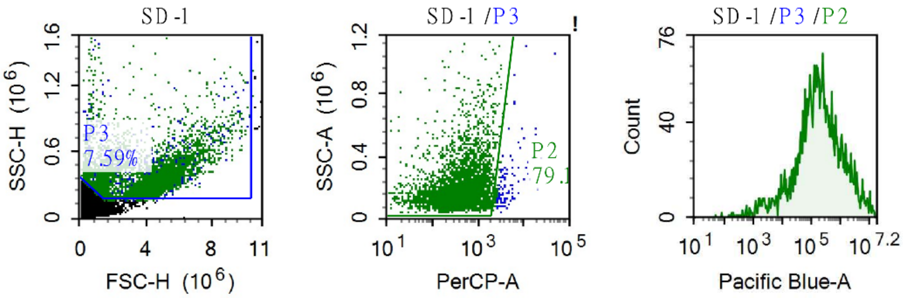

Gate	Median X	Median Y	Gate	Median X	Median Y	Gate	Median X
All	21,709	6,640	P3	330	171,402	P2	198,196
P3	4,287,942	365,410	P2	431	171,667		
			P4	0	0		

Supplementary Figure 2. Representative flow cytometry plots showing the gating strategy for the HEK293/hACE2 stable cell line binding assays.

a

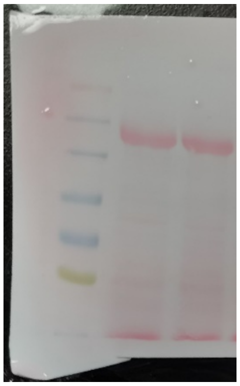

b

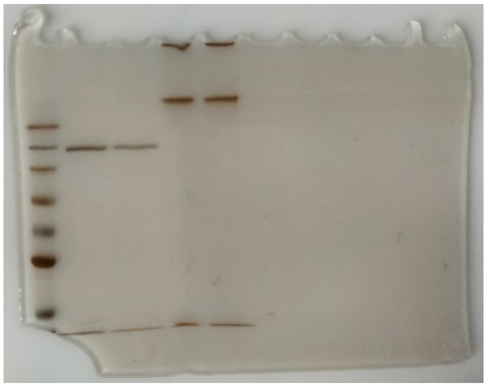

c

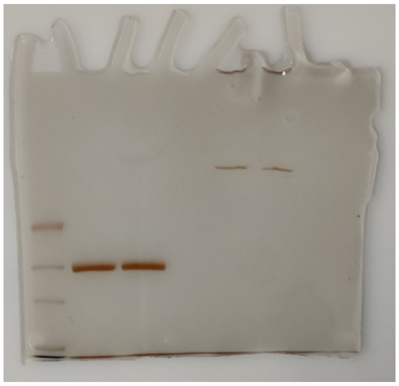

Supplementary Figure 3. Unprocessed original images. **(a)**Unprocessed original image for figure 1 b. **(b)**Unprocessed original image for figure 1 c, PF-D-Trimer. **(c)**Unprocessed original image for figure 1 c, PF-W-Trimer.
