# Supplementary material for: PF-D-Trimer, a protective SARS-CoV-2 subunit vaccine: immunogenicity and application
Source: NPJ Vaccines. 2023 Mar 15;8:38. doi: 10.1038/s41541-023-00636-8 (PMC10015519; doi:10.1038/s41541-023-00636-8)
Supplement: Supplementary file 2 — REPORTING SUMMARY [file 41541_2023_636_MOESM2_ESM.pdf]

nature portfolio

Corresponding author(s): Binlei Liu, Yan Xu, Bo Zhang, Kun Cai

Last updated by author(s): Feb 21, 2023

Reporting Summary

Nature Portfolio wishes to improve the reproducibility of the work that we publish. This form provides structure for consistency and transparency in reporting. For further information on Nature Portfolio policies, see our Editorial Policies and the Editorial Policy Checklist.

Statistics

For all statistical analyses, confirm that the following items are present in the figure legend, table legend, main text, or Methods section.

n/a	Confirmed	
☐	☒	The exact sample size (*n*) for each experimental group/condition, given as a discrete number and unit of measurement
☐	☒	A statement on whether measurements were taken from distinct samples or whether the same sample was measured repeatedly
☐	☒	The statistical test(s) used AND whether they are one- or two-sided<br>*Only common tests should be described solely by name; describe more complex techniques in the Methods section.*
☒	☐	A description of all covariates tested
☐	☒	A description of any assumptions or corrections, such as tests of normality and adjustment for multiple comparisons
☐	☒	A full description of the statistical parameters including central tendency (e.g. means) or other basic estimates (e.g. regression coefficient) AND variation (e.g. standard deviation) or associated estimates of uncertainty (e.g. confidence intervals)
☐	☒	For null hypothesis testing, the test statistic (e.g. *F*, *t*, *r*) with confidence intervals, effect sizes, degrees of freedom and *P* value noted<br>*Give P values as exact values whenever suitable.*
☒	☐	For Bayesian analysis, information on the choice of priors and Markov chain Monte Carlo settings
☒	☐	For hierarchical and complex designs, identification of the appropriate level for tests and full reporting of outcomes
☒	☐	Estimates of effect sizes (e.g. Cohen's *d*, Pearson's *r*), indicating how they were calculated

Our web collection on statistics for biologists contains articles on many of the points above.

Software and code

Policy information about availability of computer code

Data collection	*Provide a description of all commercial, open source and custom code used to collect the data in this study, specifying the version used OR state that no software was used.*
Data analysis	*Provide a description of all commercial, open source and custom code used to analyse the data in this study, specifying the version used OR state that no software was used.*

For manuscripts utilizing custom algorithms or software that are central to the research but not yet described in published literature, software must be made available to editors and reviewers. We strongly encourage code deposition in a community repository (e.g. GitHub). See the Nature Portfolio guidelines for submitting code & software for further information.

Data

Policy information about availability of data

All manuscripts must include a data availability statement. This statement should provide the following information, where applicable:

- Accession codes, unique identifiers, or web links for publicly available datasets
- A description of any restrictions on data availability
- For clinical datasets or third party data, please ensure that the statement adheres to our policy

Correspondence and requests for materials should be addressed to Yan Xu.

Human research participants

Policy information about studies involving human research participants and Sex and Gender in Research.

Reporting on sex and gender	*Use the terms sex (biological attribute) and gender (shaped by social and cultural circumstances) carefully in order to avoid confusing both terms. Indicate if findings apply to only one sex or gender; describe whether sex and gender were considered in study design whether sex and/or gender was determined based on self-reporting or assigned and methods used. Provide in the source data disaggregated sex and gender data where this information has been collected, and consent has been obtained for sharing of individual-level data; provide overall numbers in this Reporting Summary. Please state if this information has not been collected. Report sex- and gender-based analyses where performed, justify reasons for lack of sex- and gender-based analysis.*
Population characteristics	*Describe the covariate-relevant population characteristics of the human research participants (e.g. age, genotypic information, past and current diagnosis and treatment categories). If you filled out the behavioural & social sciences study design questions and have nothing to add here, write "See above."*
Recruitment	*Describe how participants were recruited. Outline any potential self-selection bias or other biases that may be present and how these are likely to impact results.*
Ethics oversight	*Identify the organization(s) that approved the study protocol.*

Note that full information on the approval of the study protocol must also be provided in the manuscript.

Field-specific reporting

Please select the one below that is the best fit for your research. If you are not sure, read the appropriate sections before making your selection.

☒ Life sciences ☐ Behavioural & social sciences ☐ Ecological, evolutionary & environmental sciences

For a reference copy of the document with all sections, see nature.com/documents/nr-reporting-summary-flat.pdf

Life sciences study design

All studies must disclose on these points even when the disclosure is negative.

Sample size	The sample sizes for the studies were determined from previous published studies, for example https://www.nature.com/articles/s41541-021-00324-5
Data exclusions	N/A
Replication	N/A
Randomization	Randomization
Blinding	N/A

Reporting for specific materials, systems and methods

We require information from authors about some types of materials, experimental systems and methods used in many studies. Here, indicate whether each material, system or method listed is relevant to your study. If you are not sure if a list item applies to your research, read the appropriate section before selecting a response.

Materials & experimental systems

n/a	Involved in the study	
☐	☒	Antibodies
☐	☒	Eukaryotic cell lines
☒	☐	Palaeontology and archaeology
☐	☒	Animals and other organisms
☒	☐	Clinical data
☒	☐	Dual use research of concern

Methods

n/a	Involved in the study	
☒	☐	ChIP-seq
☐	☒	Flow cytometry
☒	☐	MRI-based neuroimaging

Antibodies

Antibodies used	horseradish peroxidase-conjugated goat anti-mouse IgG (Proteintech, SA00001-1), goat anti-hamster IgG (Shanghai Universal Biotech Co., Ltd., 127-035-160), anti mouse IFN-γ, Mabtech, 3321-4APW-2, anti hamster IFN-γ, Mabtech 3102-4APW-2, Anti-TD monoclonal antibody, proprietary.
Validation	According to the manufacturer.

Eukaryotic cell lines

Policy information about cell lines and Sex and Gender in Research

Cell line source(s)	CHOK1-GenS, Vero E6 cells, HEK293/hACE2
Authentication	CHOK1-GenS are a commercial cell line from Genscript.
Mycoplasma contamination	Negative
Commonly misidentified lines (See ICLAC register)	*Name any commonly misidentified cell lines used in the study and provide a rationale for their use.*

Animals and other research organisms

Policy information about studies involving animals; ARRIVE guidelines recommended for reporting animal research, and Sex and Gender in Research

Laboratory animals	K18-hACE2 H11 (C57BL/6JGpt), Sprague-Dawley rats, C57BL/6 mice, Syrian hamsters
Wild animals	N/A
Reporting on sex	N/A
Field-collected samples	N/A
Ethics oversight	All animal experiments were carried out in strict accordance with the requirements of the Hubei Provincial Committee for the Management and Use of Laboratory Animals.

Note that full information on the approval of the study protocol must also be provided in the manuscript.

Flow Cytometry

Plots

Confirm that:

☒ The axis labels state the marker and fluorochrome used (e.g. CD4-FITC).

☒ The axis scales are clearly visible. Include numbers along axes only for bottom left plot of group (a 'group' is an analysis of identical markers).

☒ All plots are contour plots with outliers or pseudocolor plots.

☒ A numerical value for number of cells or percentage (with statistics) is provided.

Methodology

Sample preparation	For the cell-binding assays, 50000 HEK293/hACE2 cells were incubated at 4oC for two hours with varying concentrations of dissociated PF-D-Trimer. After the incubation, the cells were spun down at 800×g for 5 min at 4°C and washed with 1 mL of cold PBS three times in total. The cells were then incubated with a 10 µg/mL dilution of iFluor 405 labelled anti-TD antibody for one hour at 4oC. After the incubation, the cells were spun down at 800×g for 5 min at 4°C and washed with 1 mL of cold PBS three times in total. The labelled cells were analyzed with a NovoCyte 3000 Flow Cytometer (Agilent). The mean fluorescence intensities were fitted in GraphPad Prism using a specific binding with Hill slope equation.
Instrument	NovoCyte 3000 Flow Cytometer (Agilent)
Software	NovoExpress
Cell population abundance	As this is a binding assay, we analyzed the entire viable cell population to determine the median fluorescence signal.

Gating strategy

Selection of total cells in SSC/FSC, selection of viable cells after staining with 7-AAD, measurement of fluorescence in the Pacific Blue channel.

☒ Tick this box to confirm that a figure exemplifying the gating strategy is provided in the Supplementary Information.
